# Supplementary material for: EZHIP constrains Polycomb Repressive Complex 2 activity in germ cells
Source: Nat Commun. 2019 Aug 26;10:3858. doi: 10.1038/s41467-019-11800-x (PMC6710278; doi:10.1038/s41467-019-11800-x)
Supplement: Supplementary file 1 — Supplementary Information [file 41467_2019_11800_MOESM1_ESM.pdf]

SUPPLEMENTARY INFORMATION

EZHIP constrains Polycomb Repressive Complex 2 activity in germ cells

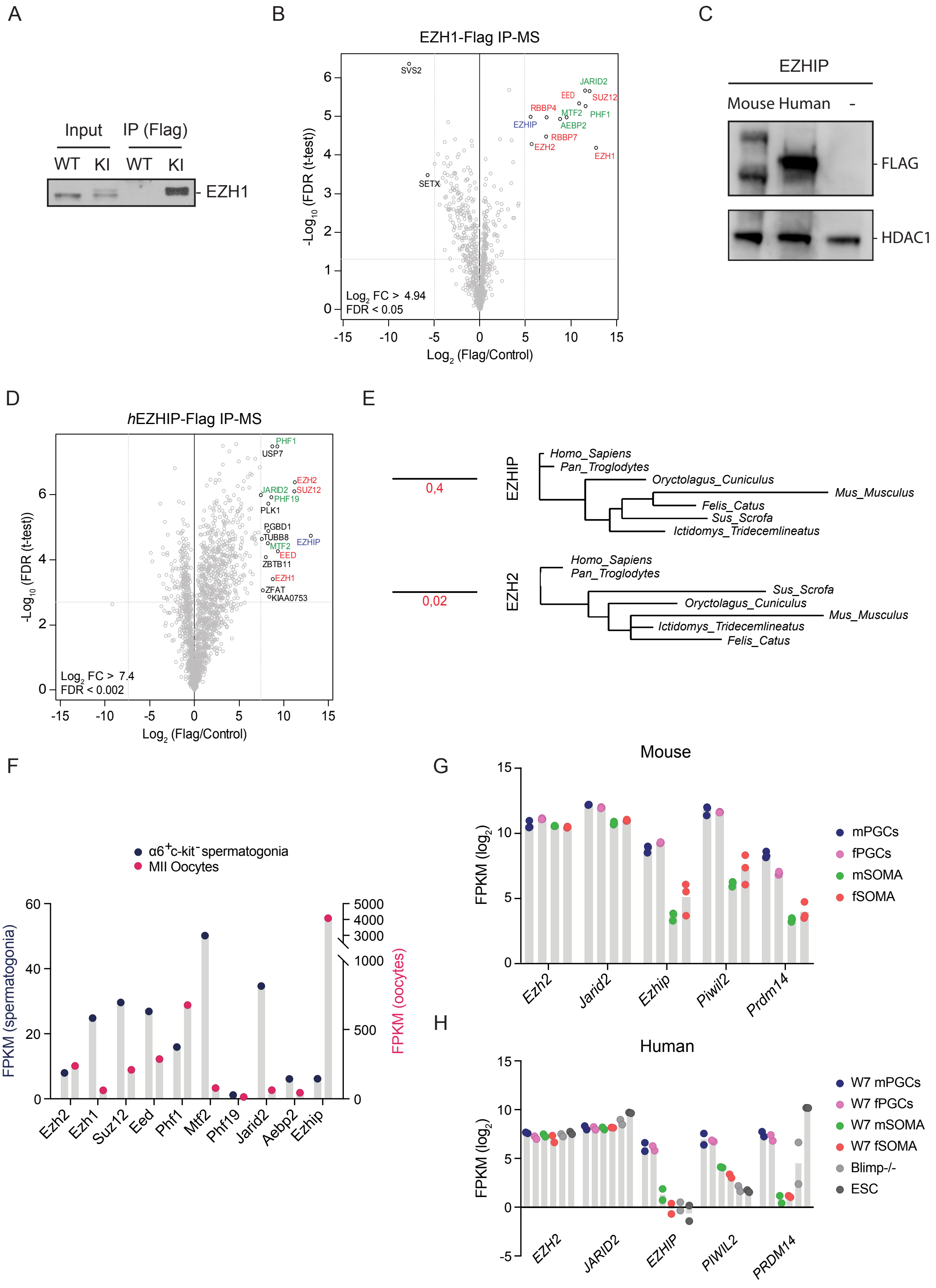

A
Input
IP (Flag)
WT
KI
WT
KI
EZH1
B
EZH1-Flag IP-MS
-Log10 (FDR (t-test))
Log2 (Flag/Control)
SVS2
JARID2
EED
SUZ12
RBBP4
MTF2
PHF1
EZHIP
AEBP2
RBBP7
EZH2
EZH1
SETX
Log2 FC > 4.94
FDR < 0.05
C
EZHIP
Mouse Human -
FLAG
HDAC1
D
hEZHIP-Flag IP-MS
-Log10 (FDR (t-test))
Log2 (Flag/Control)
PHF1
USP7
EZH2
SUZ12
JARID2
PHF19
PLK1
PGBD1
TUBB8
MTF2
EZHIP
EED
ZBTB11
EZH1
ZFAT
KIAA0753
Log2 FC > 7.4
FDR < 0.002
E
0,4
EZHIP
Homo_Sapiens
Pan_Troglodytes
Oryctolagus_Cuniculus
Mus_Musculus
Felis_Catus
Sus_Scrofa
Ictidomys_Tridecemlineatus
0,02
EZH2
Homo_Sapiens
Pan_Troglodytes
Sus_Scrofa
Oryctolagus_Cuniculus
Mus_Musculus
Ictidomys_Tridecemlineatus
Felis_Catus
F
α6+c-kit- spermatogonia
MII Oocytes
FPKM (spermatogonia)
FPKM (oocytes)
Ezh2
Ezh1
Suz12
Eed
Phf1
Mtf2
Phf19
Jarid2
Aebp2
Ezhip
G
Mouse
FPKM (log2)
Ezh2
Jarid2
Ezhip
Piwil2
Prdm14
mPGCs
fPGCs
mSOMA
fSOMA
H
Human
FPKM (log2)
EZH2
JARID2
EZHIP
PIWIL2
PRDM14
W7 mPGCs
W7 fPGCs
W7 mSOMA
W7 fSOMA
Blimp-/-
ESC

Supplementary Figure 1

(A) Western blot on nuclear extracts and Flag-IP from WT or EZH1-Flag mouse testes probed with anti-EZH1, Representative result, n=2. (B) Volcano plot representation of EZH1 interactome from EZH1-Flag mice testes IP compared to WT. In red are all the core complex subunits, in green the cofactors and in blue EZHIP, n=3. (C) Anti-FLAG western blot analysis on nuclear extracts from Hela-S3 cells stably overexpressing *Mus Musculus* or *Homo Sapiens* EZHIP and corresponding control; HDAC1 is used as a loading control, representative result. (D) Volcano plot representation of hEZHIP interactome after Flag-IP on HeLa-S3 overexpressing a tagged version of the protein. Same color code as in (B), additional interactors in black, (n=3). (E) Phylogenetic tree representing EZHIP protein sequence across placental mammals (Phylogenetic Analysis by Maximum Likelihood (PAML) algorithm). (F) PRC2 components and cofactors expression in mouse MII oocytes and α6+c-kit- spermatogonia, data extracted from the RNA-seq presented in Fig 4C and 5F (mean, n=2). (G) PRC2 components and cofactors expression in mouse somatic and germ cells [1] (GSE89711). m: male, f: female, PGC: primordial germ cells, SOMA: Somatic cells (mean, n= 3). (H) Expression of *EZHIP*, *PIWIL2*, *EZH2*, *JARID2* in human PGCs and somatic cells at week 7 [2] (NCBI SRA: SRP057098; mean, n≥ 3).

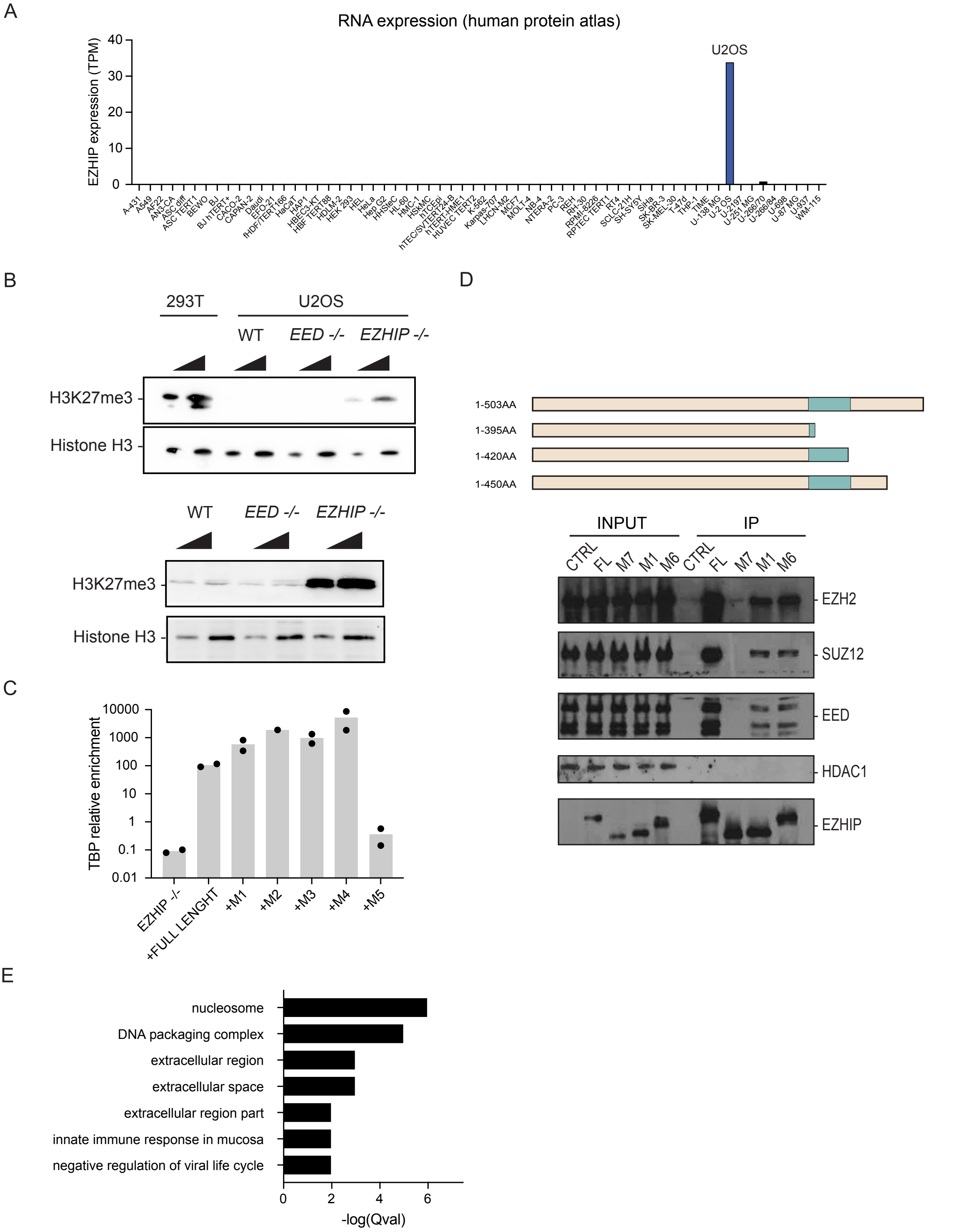

Supplementary Figure 2

(A) Human Protein Atlas cell lines transcripts analysis (TPM: transcript per million). (B) Western blot analysis of H3K27me3 and H3 (loading control) on 293T nuclear extract and U2OS nuclear extracts WT, *EED* -/- and *EZHIP* -/- (top panel). Bottom panel, same as above for U2OS extracts but loaded

with more proteins to detect H3K27me3 in WT condition, representative result. (C) RT-qPCR to detect the overexpression of *EZHIP* mutants in U2OS (control for Fig 2C, mean, n=2). (D) Different *EZHIP* C-ter truncations were expressed in HEK-293 cells (top panel). Co-IP (Flag-IP) was analyzed by western blot with the specific antibodies indicated on the right (bottom panel) (representative result, n=2). (E) GO terms the most enriched according to lowest q-value for the 287 genes found downregulated upon deletion of *EZHIP* as shown in Fig 2D. Only GO terms with a qval≤0,05 are represented.

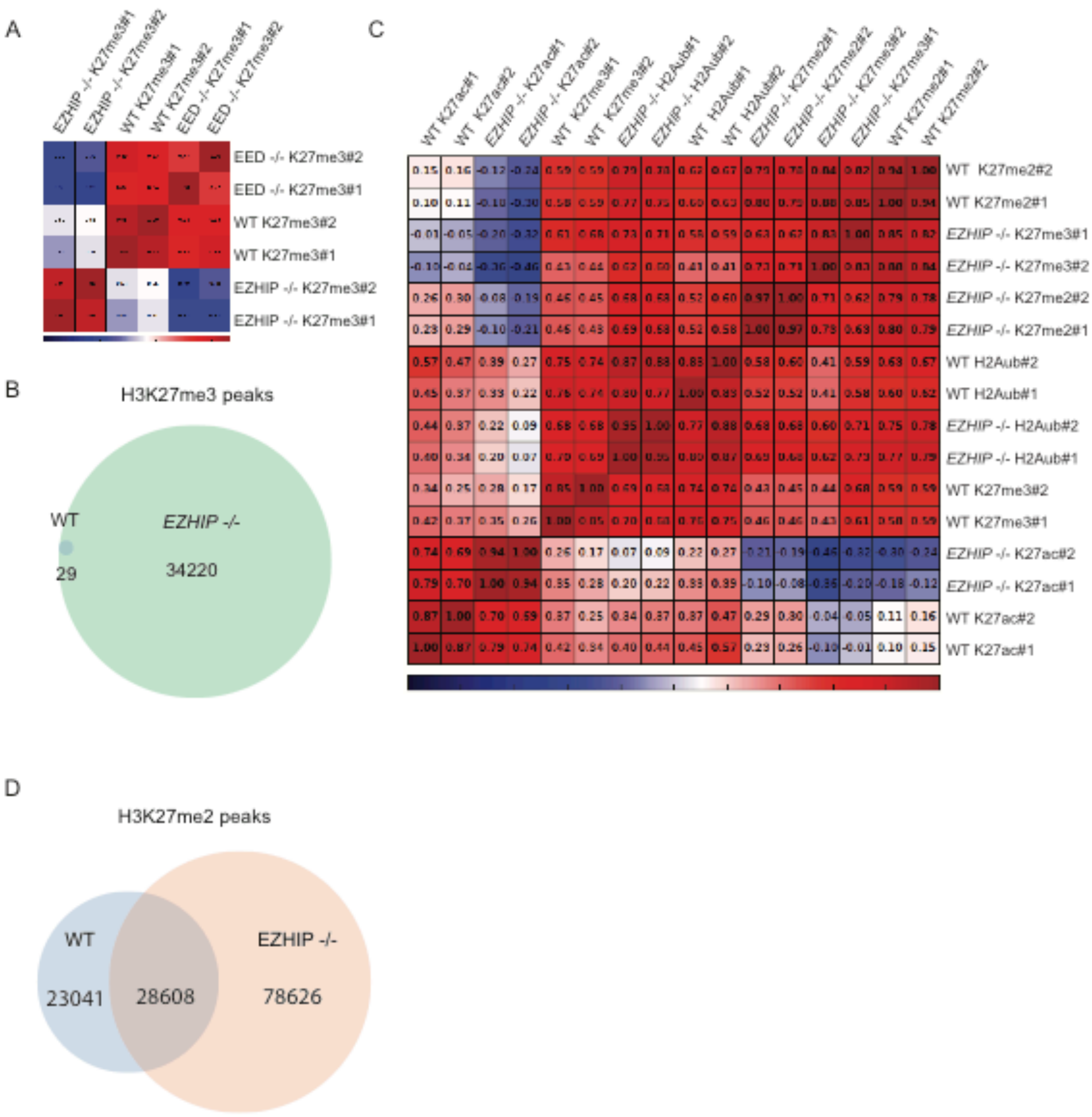

Supplementary Figure 3

(A) Correlation heatmap for H3K27me3 ChIP-seq in U2OS WT, *EED* -/- and *EZHIP* -/- (B) Venn diagram representing the peaks detected for H3K27me3 ChIP-seq in U2OS WT *versus* U2OS *EZHIP* -/-. (C) Correlation matrix for H3K27ac, H3K27me2 and H2Aub “Cut and Run” results in U2OS WT and U2OS *EZHIP* -/-. (D) Venn diagram representing the peaks detected for H3K27me2 ChIP-seq in U2OS WT *versus* U2OS *EZHIP* -/-.

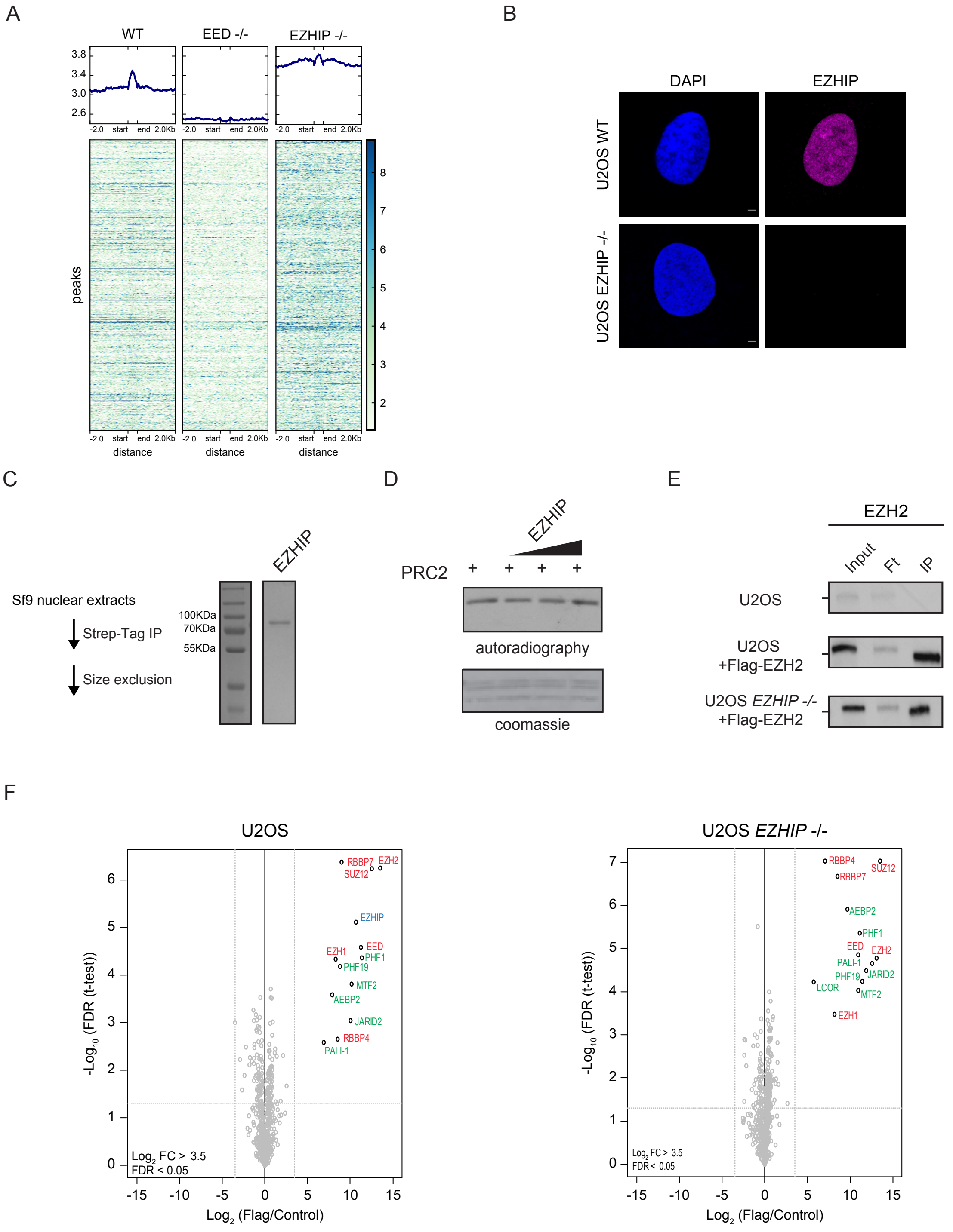

Supplementary Figure 4

(A) Heatmap representing SUZ12 enrichment in U2OS WT, *EED* -/- and *EZHIP* -/- at peaks that gain H3K27me3 enrichment in the absence of EZHIP, merged of duplicates. (B) Immunofluorescence staining for EZHIP in U2OS WT *versus* U2OS *EZHIP* -/-, nucleus stained with DAPI, representative result. Scale bars, 2 µm. (C) Left: Scheme for hEZHIP purification from Sf-9 insect cells. Right:

Coomassie staining of purified protein, representative result. (D) HKMT assay performed with rPRC2-EZH2 on native nucleosomes purified from HeLa cells in presence of increasing amount of EZHIP, n≥2. (E) Flag-IPs on extracts from U2OS (WT, WT + Flag-EZH2, *EZHIP* -/- + Flag-EZH2) analyzed by western blot and probed with an antibody recognizing EZH2, representative result. (F) Volcano plot representation of normalized mass spectrometry data after Flag-IP in U2OS+ Flag-EZH2 [3], U2OS *EZHIP* -/- Flag-EZH2 (Right), same color code as in Fig.1 and S1, n=3.

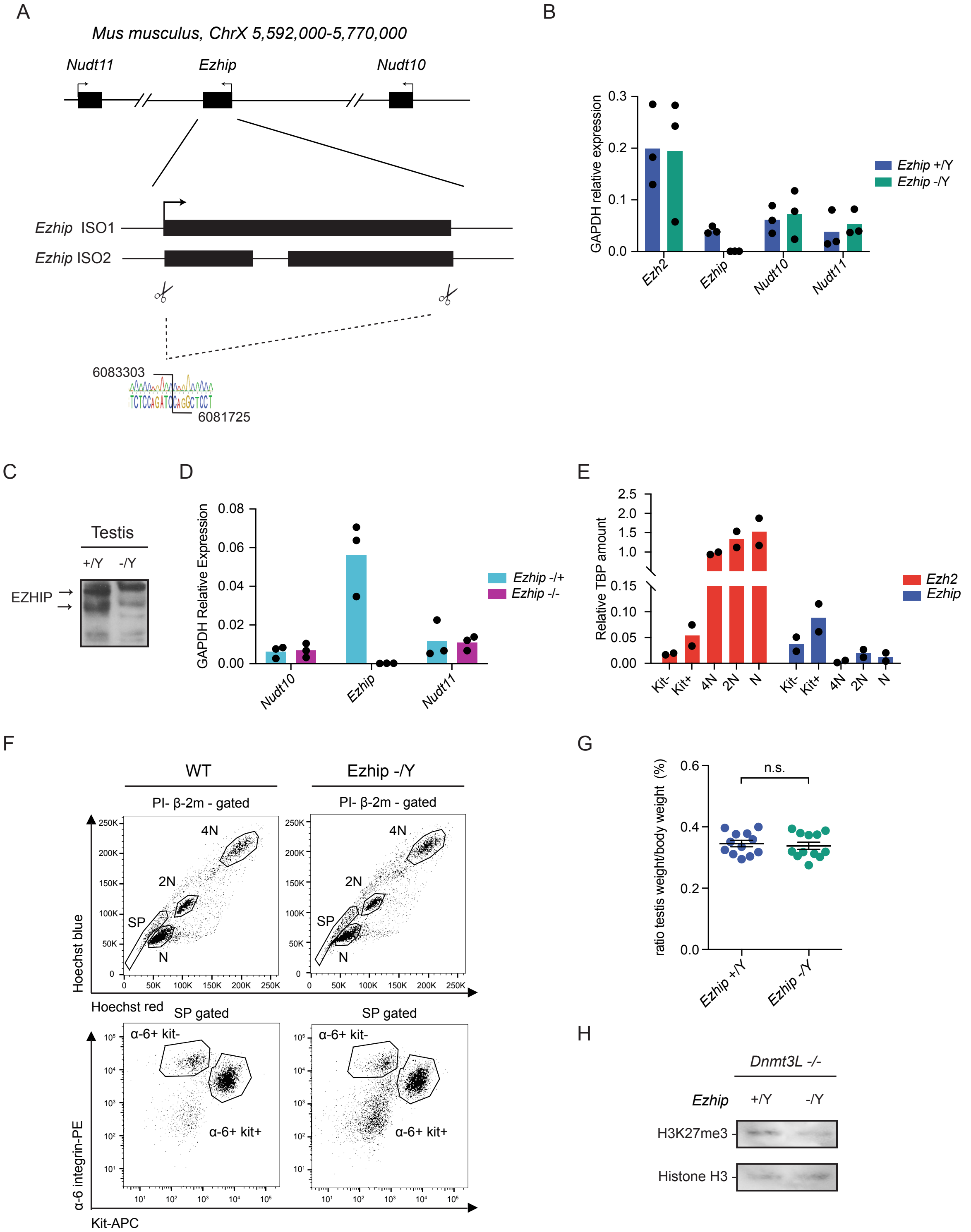

A
Mus musculus, ChrX 5,592,000-5,770,000
Nudt11
Ezhip
Nudt10
Ezhip ISO1
Ezhip ISO2
6083303
6081725
B
GAPDH relative expression
Ezhip +/Y
Ezhip -/Y
Ezh2
Ezhip
Nudt10
Nudt11
C
Testis
+/Y
-/Y
EZHIP
D
GAPDH Relative Expression
Ezhip -/+
Ezhip -/-
Nudt10
Ezhip
Nudt11
E
Relative TBP amount
Ezh2
Ezhip
Kit-
Kit+
4N
2N
N
F
WT
Ezhip -/Y
PI- β-2m - gated
4N
2N
SP
N
Hoechst blue
Hoechst red
SP gated
α-6+ kit-
α-6+ kit+
α-6 integrin-PE
Kit-APC
G
ratio testis weight/body weight (%)
n.s.
Ezhip +/Y
Ezhip -/Y
H
Dnmt3L -/-
Ezhip
+/Y
-/Y
H3K27me3
Histone H3

Supplementary Figure 5

(A) Schematic representation of *Ezhip* locus, scissors indicate the deletion generated by genome editing in mice. At the bottom is a representative sequencing of this locus in founders. (B) *Nudt10*, *Ezhip*, *Nudt11* and *Ezh2* mRNA relative abundancy normalized to *Gapdh* in whole testis from adult male mice WT and *Ezhip* -/-, mean, n= 3. (C) WB analysis with mouse anti-EZHIP on WT and *Ezhip* -/- testis nuclear extracts. Arrows indicate specific signal, representative result. (D) *Nudt10*, *Ezhip* and *Nudt11* mRNA normalized to *Gapdh* mRNA in whole ovaries from adult female mice WT and *Ezhip* -/-, mean, n= 3. (E) RT-qPCR analysis of *Ezhip* and *Ezh2* mRNA expression during spermatogenesis. *Ezhip* and *Ezh2* mRNA are normalized to *Tbp*. The different spermatogenic populations (undifferentiated spermatogonia kit-, differentiating spermatogonia kit+, 4N, 2N, N) have been sorted by FACS, mean, n= 2. (F) Analysis by flow cytometry of testicular cell suspensions from WT and *Ezhip* -/Y mice: spermatocyte I (4N), spermatocyte II (2N), spermatids (N), and differentiating (α-6^{+} kit^{+}) and undifferentiated spermatogonia (α-6^{+} kit^{-}). α-6 (α-6 integrin), kit (c-kit receptor), PI (propidium iodide), β-2m (β-2 microglubulin), and SP (Side Population) markers are indicated in graphs, representative result. (G) Mice testis absolute weight (mg), mean ±sem, n=12. Significance: unpaired, non-parametric test of Kologorov-Smirnov. (H) Western blot analysis of H3K27me3 and H3 levels on whole testis extracts of WT; *Dnmt3l* -/- and *Ezhip* -/-; *Dnmt3 l*-/- mice. Representative result.

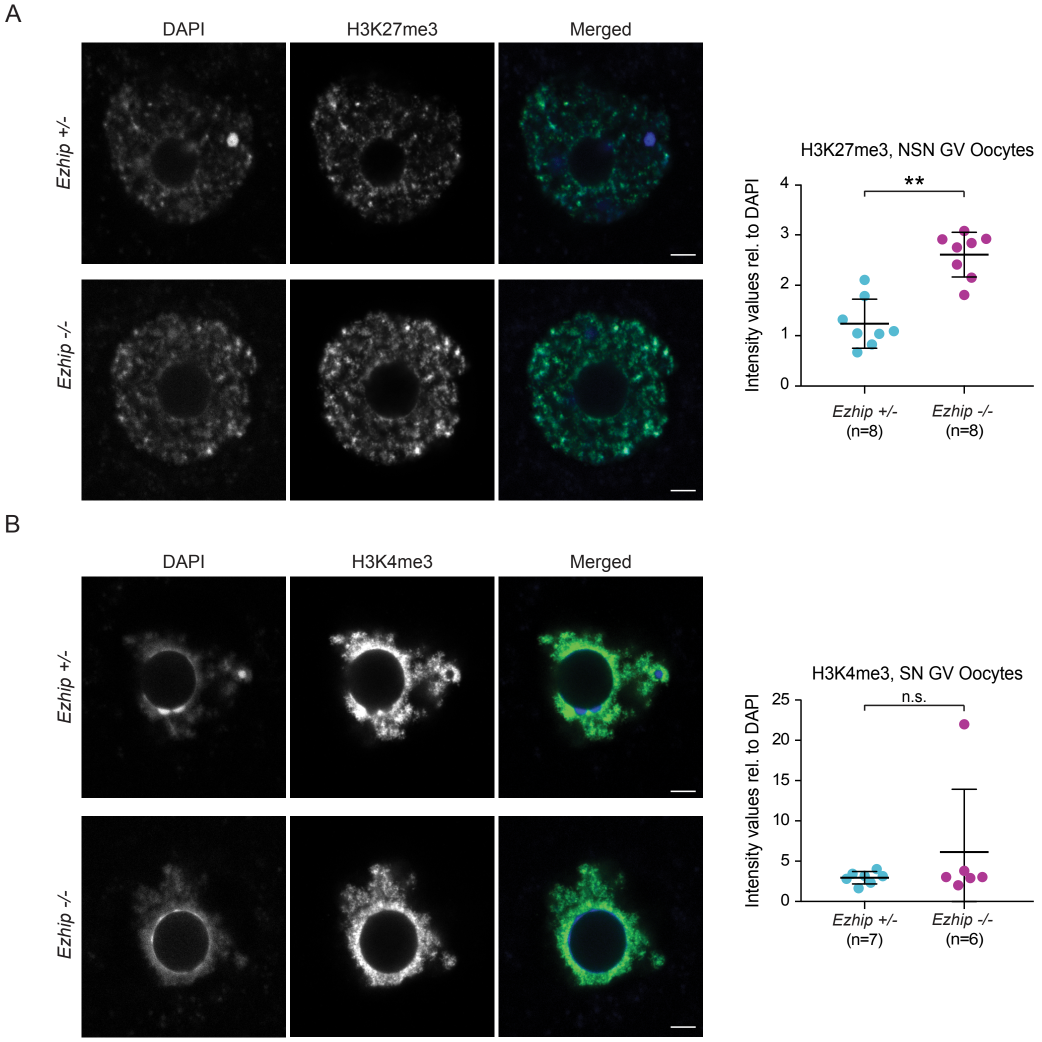

Supplementary Figure 6

(A) Non-surrounded Nuclei (NSN) WT and *Ezhip* -/- oocytes were fixed and stained for H3K27me3 (green in merged). DNA was stained with DAPI (blue in merged). IF quantification is indicated on the right, H3K27me3 intensities are normalized to DAPI. Scale bars, 5 µm. (B) Same as in (A) but Surrounded Nuclei (SN) oocytes were probed for H3K4me3. Scale bars, 5 µm. (A, B) mean ± s.d., each dot represents a follicle, *n* indicated on the graph. Significance: unpaired, non-parametric test of Kologorov-Smirnov, $^{**}P \leq 0.01$.

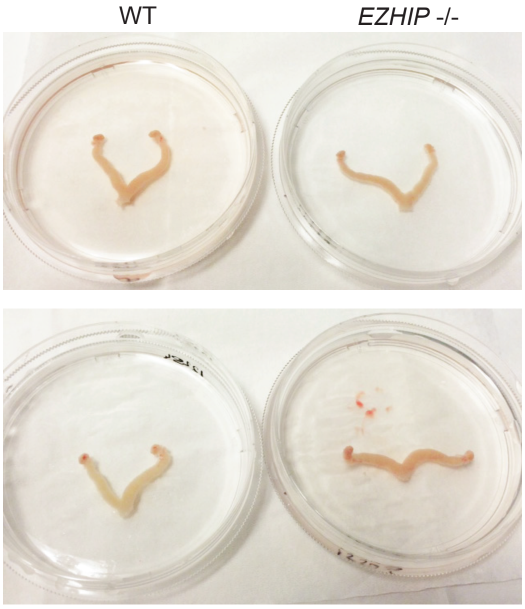

Supplementary Figure 7

Images showing representative reproductive track of 6-weeks-old WT and *Ezhip* -/- females.

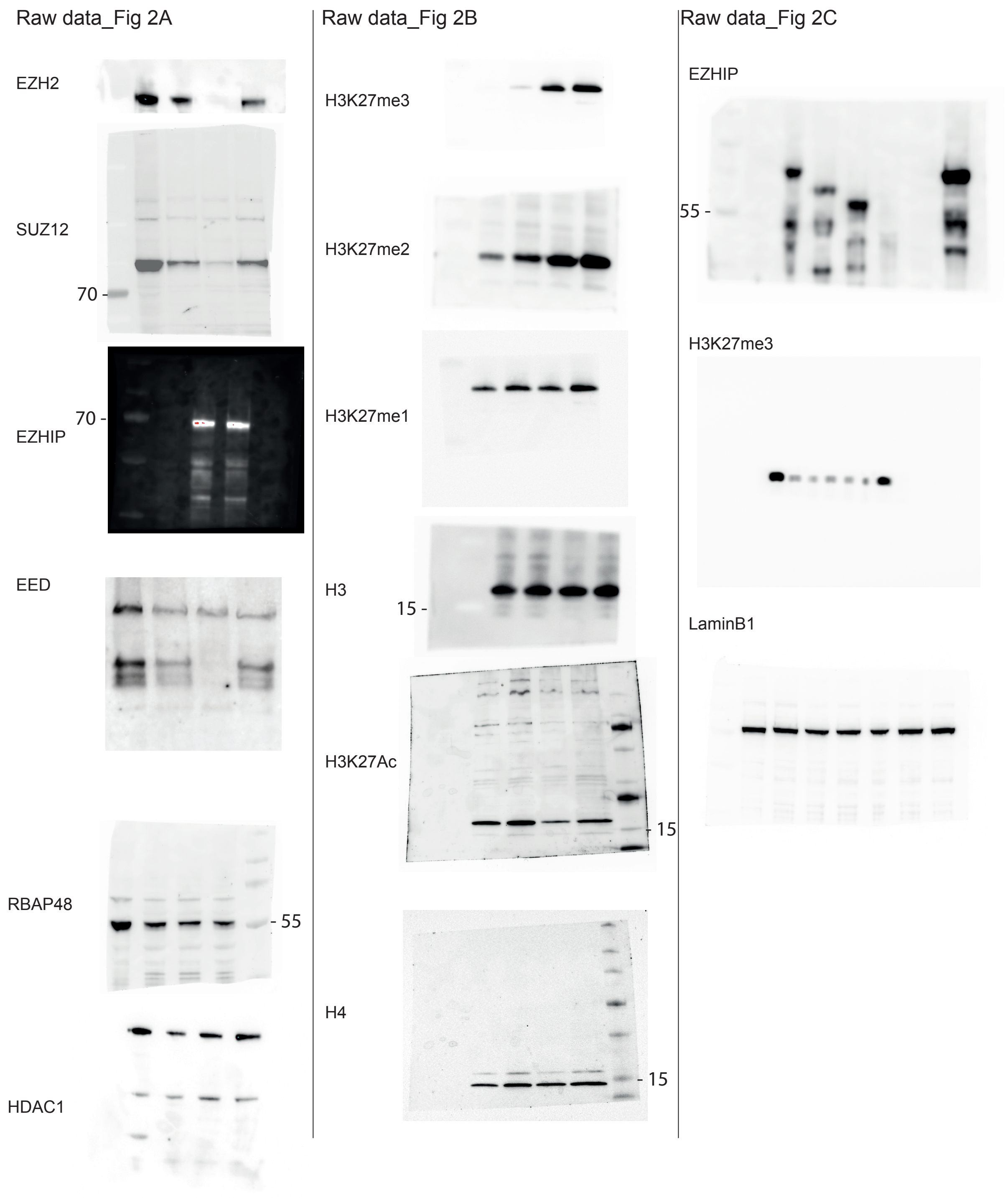

Supplementary Figure 8
Raw data for Figure 2A, 2B & 2C

Raw data_Fig 4C

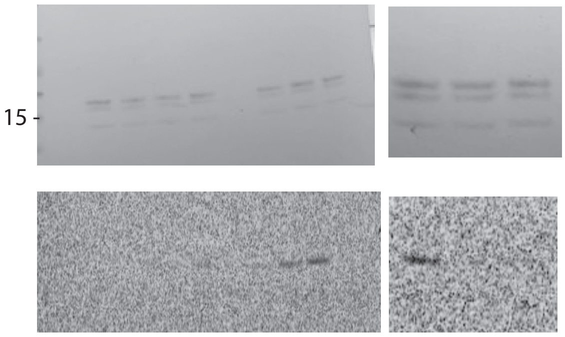

Raw data_Fig 4E

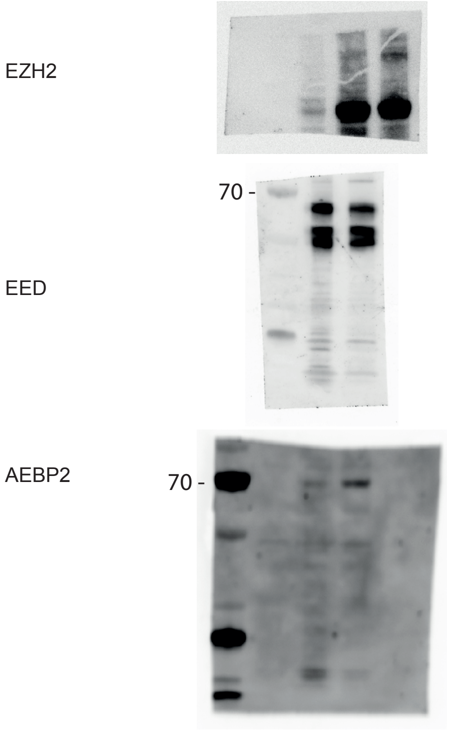

Supplementary Figure 9

Raw data for Figure 4C & 4E

Supplementary Figure 10
Raw data for Figure 5A & 5F

Raw data_Fig S1A

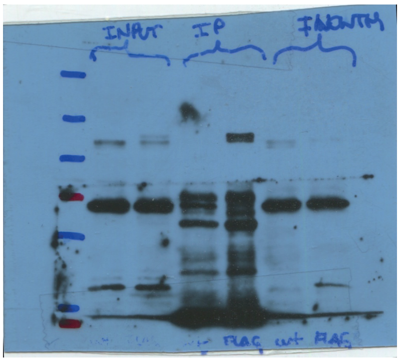

Raw data_Fig S1C

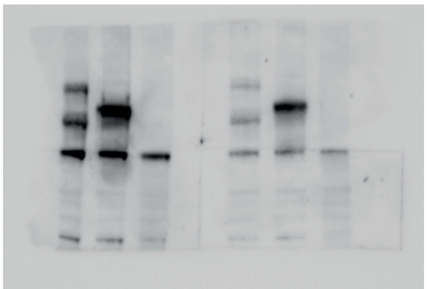

Raw data_Fig S1A

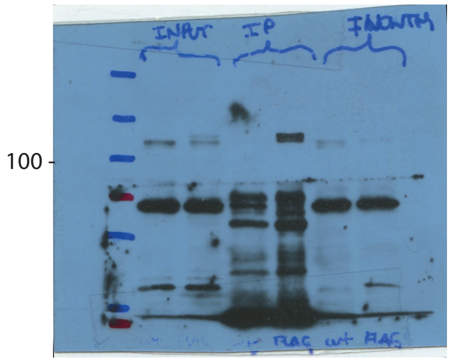

Raw data_Fig S1C

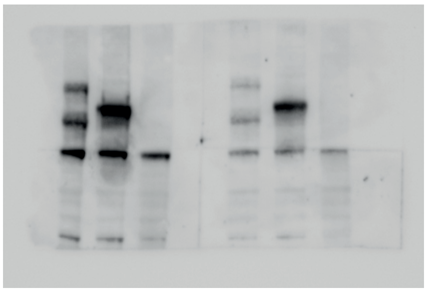

Supplementary Figure 11
Raw data for Supplementary Figure 1A & 1C

Raw data_Fig S2B

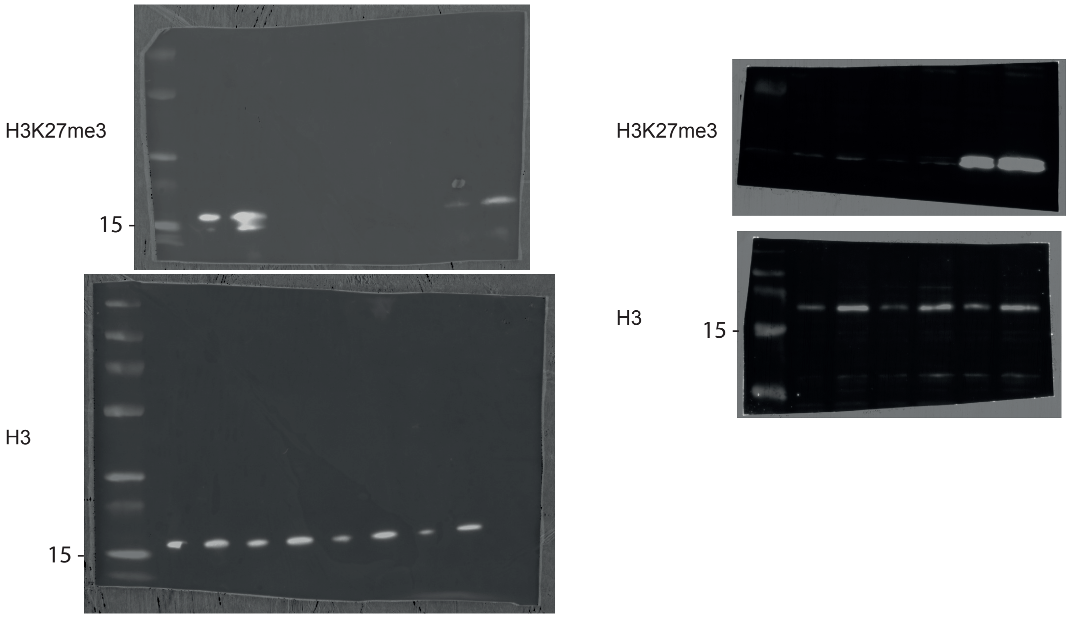

Raw data_Fig S2D

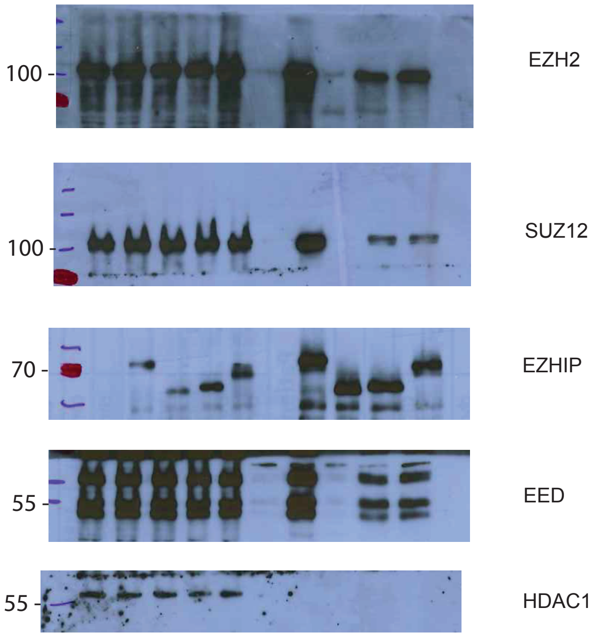

Supplementary Figure 12
Raw data for Supplementary Figure 2B & 2D

Raw data_Fig S4C

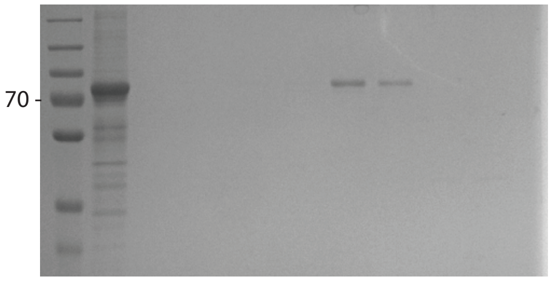

Raw data_Fig S4D

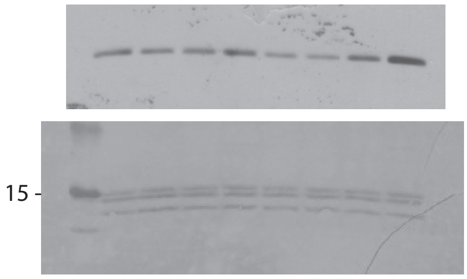

Raw data_Fig S4E

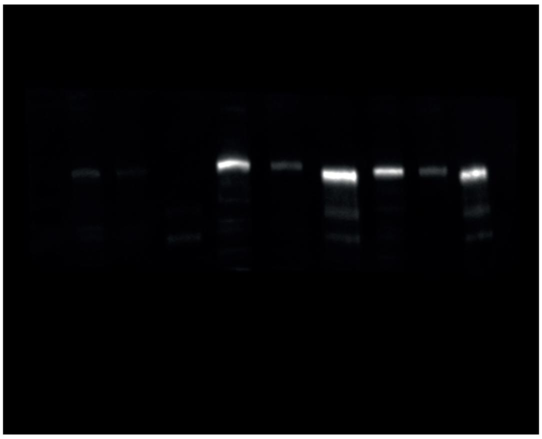

Supplementary Figure 13
Raw data for Supplementary Figure 4C, 4D & 4E

Raw data_Fig S5C

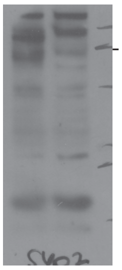

Raw data_Fig S5H

H3K27me3

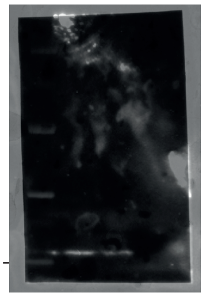

15 -

H3

15 -

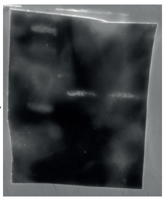

Supplementary Figure 14
Raw data for Supplementary Figure 5C & 5H

Supplementary Table 1

Full analysis of computer-assisted spermatozoa images.
In red are the numbers represented in Fig 5E.

General Characteristics	KOY	KOY	WTY	WTY
Path velocity	140,8	147,5	135,5	132,5
Prog. Velocity	109	110,6	106,7	97,8
Track speed	251,3	280,6	243,6	255
Lateral amplitude	10,3	11,4	10	10,9
Beat frequency	28,2	28,8	28,4	30,6
Stranghtness	77	74	77	74
Linearity	47	41	47	40
Elongation	53	54	53	51
Area	47,1	48,7	47,3	54
**Sperm properties**				
Total sperm cells counted	428	868	595	1311
Motile	259	631	426	1140
Progressive	143	311	249	525
Expressed as Percentage of the total %	100	100	100	100
	61	73	72	87
	43	36	42	42
**Sperm speed**				
Rapid	243	599	415	1096
Medium	16	32	11	44
Slow	3	4	4	8
Static	166	233	165	163
Expressed as Percentage of the total %	57	69	70	84
	4	4	2	3
	1	0	1	1
	39	27	28	12

Supplementary Table 2

Primers sequences.

Name	Application	Sequence
mGapdh FW	RT-qPCR	AACAGCAACTCCCACTCTTC
mGapdh REV	RT-qPCR	TGGTCCAGGGTTTCTTACTC
mEzh2 FW	RT-qPCR	AATACATGTGCAGCTTTCTGTTC
mEzh2 REV	RT-qPCR	ACGAATTTTGTTGCCCTTTC
mEzhip FW	RT-qPCR	TTCCGGAGTTGTACCTTTCG
mEzhip REV	RT-qPCR	ACGTAAATTCCAGCCTGTGC
mNudt10 FW	RT-qPCR	AGAGAGCGAGCCCTAGTGAATGGA
mNudt10 REV	RT-qPCR	GAGCTCACCTGTGCTTCACAATTCC
mNudt11 FW	RT-qPCR	ACCGAGGCATGCTCAAGATCACA
mNudt11 REV	RT-qPCR	TGAGCGGTCTCCTTGGCAACCTTA
hEZHIP N-ter FW	RT-qPCR	ACCTCCGCCGCCATTTTCATCA
hEZHIP N-ter REV	RT-qPCR	TCGGGCACCACACACCCAAAAA
hEZHIP stretch FW	RT-qPCR	GCCTGTTTGGCATGCAGTCCGTAT
hEZHIP stretch REV	RT-qPCR	ACTGCTGAGGGATGGGAAGGAAGA

Supplemental References

1. Percharde, M., Wong, P. & Ramalho-Santos, M. Global Hypertranscription in the Mouse Embryonic Germline. *Cell Rep* **19**, 1987-1996 (2017).
2. Tang, W.W. et al. A Unique Gene Regulatory Network Resets the Human Germline Epigenome for Development. *Cell* **161**, 1453-67 (2015).
3. Steger, D.J. et al. DOT1L/KMT4 recruitment and H3K79 methylation are ubiquitously coupled with gene transcription in mammalian cells. *Mol Cell Biol* **28**, 2825-39 (2008).
