# Supplementary material for: EZHIP constrains Polycomb Repressive Complex 2 activity in germ cells
Source: Nat Commun. 2019 Aug 26;10:3858. doi: 10.1038/s41467-019-11800-x (PMC6710278; doi:10.1038/s41467-019-11800-x)
Supplement: Supplementary file 3 — Reporting Summary [file 41467_2019_11800_MOESM3_ESM.pdf]

nature research

Corresponding author(s): Raphaël Margueron

Reporting Summary

Nature Research wishes to improve the reproducibility of the work that we publish. This form provides structure for consistency and transparency in reporting. For further information on Nature Research policies, see Authors & Referees and the Editorial Policy Checklist.

Statistical parameters

When statistical analyses are reported, confirm that the following items are present in the relevant location (e.g. figure legend, table legend, main text, or Methods section).

n/a	Confirmed	
☐	☒	The exact sample size (*n*) for each experimental group/condition, given as a discrete number and unit of measurement
☐	☒	An indication of whether measurements were taken from distinct samples or whether the same sample was measured repeatedly
☐	☒	The statistical test(s) used AND whether they are one- or two-sided<br>*Only common tests should be described solely by name; describe more complex techniques in the Methods section.*
☒	☐	A description of all covariates tested
☐	☒	A description of any assumptions or corrections, such as tests of normality and adjustment for multiple comparisons
☐	☒	A full description of the statistics including central tendency (e.g. means) or other basic estimates (e.g. regression coefficient) AND variation (e.g. standard deviation) or associated estimates of uncertainty (e.g. confidence intervals)
☒	☐	For null hypothesis testing, the test statistic (e.g. *F*, *t*, *r*) with confidence intervals, effect sizes, degrees of freedom and *P* value noted<br>*Give P values as exact values whenever suitable.*
☒	☐	For Bayesian analysis, information on the choice of priors and Markov chain Monte Carlo settings
☒	☐	For hierarchical and complex designs, identification of the appropriate level for tests and full reporting of outcomes
☒	☐	Estimates of effect sizes (e.g. Cohen's *d*, Pearson's *r*), indicating how they were calculated
☐	☒	Clearly defined error bars<br>*State explicitly what error bars represent (e.g. SD, SE, CI)*

Our web collection on statistics for biologists may be useful.

Software and code

Policy information about availability of computer code

Data collection	Imaging data, protein analysis have been collected with specific software described in the Material and Method section.
Data analysis	All analyses were performed using existing tools with version and parameters detailed in the methods section.

For manuscripts utilizing custom algorithms or software that are central to the research but not yet described in published literature, software must be made available to editors/reviewers upon request. We strongly encourage code deposition in a community repository (e.g. GitHub). See the Nature Research guidelines for submitting code & software for further information.

Data

Policy information about availability of data

All manuscripts must include a data availability statement. This statement should provide the following information, where applicable:

- Accession codes, unique identifiers, or web links for publicly available datasets
- A list of figures that have associated raw data
- A description of any restrictions on data availability

All information are provided in manuscript under "Data availability"

Field-specific reporting

Please select the best fit for your research. If you are not sure, read the appropriate sections before making your selection.

☒ Life sciences ☐ Behavioural & social sciences ☐ Ecological, evolutionary & environmental sciences

For a reference copy of the document with all sections, see nature.com/authors/policies/ReportingSummary-flat.pdf

Life sciences study design

All studies must disclose on these points even when the disclosure is negative.

Sample size	All experiments were repeated by analyzing independent biological replicates.<br>When comparing cell lines, experiments were done at different times with independent batches of cells.
Data exclusions	Experiments were excluded when controls (positive or negative) did not folow the expected trend.
Replication	Experiments have been repeated at least twice with variable "n" according to the type of experiment.
Randomization	n/a
Blinding	n/a

Reporting for specific materials, systems and methods

Materials & experimental systems

n/a	Involved in the study	
☒	☐	Unique biological materials
☐	☒	Antibodies
☐	☒	Eukaryotic cell lines
☒	☐	Palaeontology
☐	☒	Animals and other organisms
☒	☐	Human research participants

Methods

n/a	Involved in the study	
☐	☒	ChIP-seq
☐	☒	Flow cytometry
☒	☐	MRI-based neuroimaging

Antibodies

Antibodies used

Antibodies against EZH1/2, SUZ12, JARID2 and EED have been previously described 33,61.
RBAP48 mouse mAb (GWB-C12FDE) was purchased GenWay Biotech;
H3 mAb (39163) and H3K27me2 mAb (61435) were purchased from Active Motif.
Polyclonal Rabbit against H3 from cell signalling (9715);
H3K9me2 (Ab1220) and H3K27Ac (Ab 4729) have been purchased from Abcam;
H3K27me1 mouse mAb C0321 from Active Motif;
H3K27me2 Rabbit mAb D18C8 (9728), H3K27me3 Rabbit mAb C36B11 (9733);
H3K4me3 Rabbit mAb C42D8 (9751), Rabbit mAb D7C6X (14129) and mouse mAb10E2 HDAC1 (5356S) from Ozyme (Cell Signaling Technology);
hGPIF (HPA006128) and mAb Flag-M2 (F1804) purchased from SIGMA;
Anti-Germ cell specific Rabbit Polyclonal DPP3A/Stella (19878) and TRA98 (Ab82527) Rat monoclonal one from Abcam.
Antibody against mGPIF was raised against the two following synthetic peptides:
CAESSRAESDQSSPAG (corresponding to a.a. 91-106) and CAQSAGRNLRPRPRSS (corresponding to a.a. 192-206).
Anti mouse CD49f-PE clone GoH3 (Becton Dickinson)
Anti mouse c-kit-APC clone 2B8 (Becton Dickinson)
Anti mouse beta-2 microglobulin-FITC clone S19.8 (Santa Cruz)
Anti-mouse β-TUBULIN was purchased from Invitrogen 32–2600

Validation

- Antibodies against EZH1/2, SUZ12, JARID2 and EED have been validated in their respective knock-out cell lines by lack of signal.

- RBAP48 mouse mAb (GWB-C12FDE) was validated on overexpressed protein.
- H3 mAb (39163) has been purchased from Active Motif and validated with recombinant protein;
- H3 polyclonal Rabbit one from cell signalling (9715) and has been validated by the manufacturer (https://www.cellsignal.com/products/primary-antibodies/histone-h3-antibody/9715);
-H3K9me2 (Ab1220) and H3K27Ac (Ab 4729) from Abcam have been validated by peptide arrays;
-H3K27me1 mouse mAb C0321 from Active Motif has been validated by peptide-array;
H3K27me2 Rabbit mAb D18C8 (9728), H3K27me3 Rabbit mAb C36B11 (9733) have been validated by lack of signal in Eed knockout cell;
-H3K4me3 Rabbit mAb C42D8 (9751), validation by manufacturer (https://www.cellsignal.com/products/primary-antibodies/tri-methyl-histone-h3-lys4-c42d8-rabbit-mab/9751))
- mAb10E2 HDAC1 (5356S) from Ozyme (Cell Signaling Technologies) has been validated by the manufacturer (https://www.cellsignal.com/products/primary-antibodies/hdac1-10e2-mouse-mab/5356);
- hGPIF (HPA006128) has been validated in the GPIF knockout line
- mAb Flag-M2 (F1804, Sigma), validation by protein overexpression;
- Anti-Germ cell specific rabbit Polyclonal DPP3A/Stella (19878) validated by Abcam (https://www.abcam.com/stella-antibody-ab19878.html);
- TRA98 (Ab82527) Rat monoclonal has been validated by Abcam (https://www.abcam.com/germ-cell-specific-antigen-antibody-tra98-ab82527.html);
- Antibody against mGPIF was raised against the two following synthetic peptides and validated in the knockout extracts.
- Anti mouse CD49f-PE clone GoH3 (Becton Dickinson) has been validated by the manufacturer (http://www.bdbiosciences.com/eu/applications/research/stem-cell-research/cancer-research/human/pe-rat-anti-human-cd49f-goh3/p/555736)
- Anti mouse c-kit-APC clone 2B8 (Becton Dickinson) has been validated by the manufacturer (http://www.bdbiosciences.com/eu/applications/research/stem-cell-research/cancer-research/mouse/apc-rat-anti-mouse-cd117-2b8/p/561074)
- Anti mouse beta-2 microglobulin-FITC clone S19.8 (Santa Cruz) has been validated by the manufacturer (https://www.scbt.com/scbt/product/beta-2-microglobulin-antibody-s19-8)
Anti-mouse β-TUBULIN 32–2600 was validated by Invitrogen

Eukaryotic cell lines

Policy information about cell lines

Cell line source(s)

U2OS were purchased from ATCC; Hela-S3 provided By S. Ait-Si-Ali.

Authentication

None of the cell lines used were authenticated.

Mycoplasma contamination

Mycoplasma tests were run on monthly base.

Commonly misidentified lines
(See ICLAC register)

n/a

Animals and other organisms

Policy information about studies involving animals; ARRIVE guidelines recommended for reporting animal research

Laboratory animals

CRISPR/Cas9 Gpif knockout mice line has been generated on a C57B6N genetic background.
Phenotype analysis has been performed after back-crossing twice the mutated line with C57B6N mice.
Adult (from 3 months old onwards) males wt have been compared to hemizygous siblings or litter mates of the same age.
P17 knockout and heterozygous litter-mates have been employed in prepubertal analysis.
4-5 months old adult females wt, heterozygous and knockout litter-mates have been used in phenotypic analysis. Females with same age have been compared.
6 weeks young wt and knockout females have been crossed in the same cage to a fertile male for 20 weeks to compare fertility.

Wild animals

n/a

Field-collected samples

n/a

ChIP-seq

Data deposition

☒ Confirm that both raw and final processed data have been deposited in a public database such as GEO.

☒ Confirm that you have deposited or provided access to graph files (e.g. BED files) for the called peaks.

Data access links
May remain private before publication.

NGS data are deposited on GEO, GSE130231 and released.

Files in database submission

GSM3734215	U2OS_WT_1	Apr 01, 2020	approved	BW
GSM3734216	U2OS_WT_2	Apr 01, 2020	approved	BW
GSM3734217	U2OS_dEED_1	Apr 01, 2020	approved	BW
GSM3734218	U2OS_dEED_2	Apr 01, 2020	approved	BW
GSM3734219	U2OS_dCXORF67_1	Apr 01, 2020	approved	BW

GSM3734220 U2OS_dCXORF67_2 Apr 01, 2020 approved BW
GSM3734221 U2OS_WT-1-IgG Apr 01, 2020 approved BW
GSM3734222 U2OS_WT-2-IgG Apr 01, 2020 approved BW
GSM3734223 U2OS_WT-1-K27ac Apr 01, 2020 approved BW
GSM3734224 U2OS_WT-2-K27ac Apr 01, 2020 approved BW
GSM3734225 U2OS_WT-1-K27me2 Apr 01, 2020 approved BW
GSM3734226 U2OS_WT-2-K27me2 Apr 01, 2020 approved BW
GSM3734227 U2OS_WT-1-H2A-ub Apr 01, 2020 approved BW
GSM3734228 U2OS_WT-2-H2A-ub Apr 01, 2020 approved BW
GSM3734229 U2OS_ko-1-IgG Apr 01, 2020 approved BW
GSM3734230 U2OS_ko-2-IgG Apr 01, 2020 approved BW
GSM3734231 U2OS_ko-1-K27ac Apr 01, 2020 approved BW
GSM3734232 U2OS_ko-2-K27ac Apr 01, 2020 approved BW
GSM3734233 U2OS_ko-1-K27me2 Apr 01, 2020 approved BW
GSM3734234 U2OS_ko-2-K27me2 Apr 01, 2020 approved BW
GSM3734235 U2OS_ko-1-H2A-ub Apr 01, 2020 approved BW
GSM3734236 U2OS_ko-2-H2A-ub Apr 01, 2020 approved BW
GSM3737367 U2OS_dCXORF67_Suzcom-1 Apr 01, 2020 approved BW
GSM3737368 U2OS_dCXORF67_Suzcom_2 Apr 01, 2020 approved BW
GSM3737369 U2OS_dEED_Suzcom_1 Apr 01, 2020 approved BW
GSM3737370 U2OS_dEED_Suzcom_2 Apr 01, 2020 approved BW
GSM3737371 U2OS_WT_Suzcom_1 Apr 01, 2020 approved BW
GSM3737372 U2OS_WT_Suzcom_2 Apr 01, 2020 approved BW

GSE130229 EZHIP constrains Polycomb Repressive Complex 2 activity in germ cells (RNA-Seq) Apr 01, 2020 approved None
GSM3734182 dCXorf67cl29_1 Apr 01, 2020 approved TAB
GSM3734183 dCXorf67cl29_2 Apr 01, 2020 approved TAB
GSM3734184 dEEDcl71 Apr 01, 2020 approved TAB
GSM3734185 dEEDcl72 Apr 01, 2020 approved TAB
GSM3734186 WT1 Apr 01, 2020 approved TAB
GSM3734187 WT2 Apr 01, 2020 approved TAB
GSM3734188 WT_oocytes_2 Apr 01, 2020 approved TAB
GSM3734189 WT_oocytes_1 Apr 01, 2020 approved TAB
GSM3734190 KO_oocytes_2 Apr 01, 2020 approved TAB
GSM3734191 KO_oocytes_1 Apr 01, 2020 approved TAB
GSM3734192 KO_Kitmoins_1 Apr 01, 2020 approved TAB
GSM3734193 KO_Kitmoins_2 Apr 01, 2020 approved TAB
GSM3734194 WT_Kitmoins_1 Apr 01, 2020 approved TAB
GSM3734195 WT_Kitmoins_2 Apr 01, 2020 approved TAB
GSM3734196 46_MII_WT Apr 01, 2020 approved TAB
GSM3734197 47_MII_WT Apr 01, 2020 approved TAB
GSM3734198 48_MII_WT Apr 01, 2020 approved TAB
GSM3734199 49_MII_WT Apr 01, 2020 approved TAB
GSM3734200 50_MII_WT Apr 01, 2020 approved TAB
GSM3734201 51_MII_WT Apr 01, 2020 approved TAB
GSM3734202 52_MII_WT Apr 01, 2020 approved TAB
GSM3734203 54_MII_WT Apr 01, 2020 approved TAB
GSM3734204 55_MII_WT Apr 01, 2020 approved TAB
GSM3734205 56_MII_KO Apr 01, 2020 approved TAB
GSM3734206 57_MII_KO Apr 01, 2020 approved TAB
GSM3734207 58_MII_KO Apr 01, 2020 approved TAB
GSM3734208 59_MII_KO Apr 01, 2020 approved TAB
GSM3734209 60_MII_KO Apr 01, 2020 approved TAB
GSM3734210 61_MII_KO Apr 01, 2020 approved TAB
GSM3734211 62_MII_KO Apr 01, 2020 approved TAB
GSM3734212 63_MII_KO Apr 01, 2020 approved TAB
GSM3734213 64_MII_KO Apr 01, 2020 approved TAB
GSM3734214 65_MII_KO Apr 01, 2020 approved TAB

Genome browser session (e.g. UCSC)

see data access links for bigWig and peak BED files

Methodology

Replicates

2 biological replicates were performed for each NGS analysis.

Sequencing depth

Information provided in materials and methods

Antibodies

Information provided above in the antibody description.

Peak calling parameters

Peaks were called with MACS2 on combined replicates using the EED KO as a control for the K27me3 and SUZ12 ChIP and IgG control for CUT&RUN samples with the following parameters: -f BAM --gsize hs --broad --broad-cutoff 0.1 --bdg. Reads

	were counted in bins of length 50 and RPKM normalized and converted to bigWig format using DeepTools bamCoverage (2.4.1). Spike-in normalization: reads mapping to the drosophila genome were counted into 10kb bins and scale factors were calculated using DESeq2 estimateSizeFactors.
Data quality	Raw data was assessed using FastQC and correlation coefficients were computed for biological replicates.
Software	all analysis was performed using existing software and parameters and versions are detailed in the methods

Flow Cytometry

Plots

Confirm that:

☒ The axis labels state the marker and fluorochrome used (e.g. CD4-FITC).

☒ The axis scales are clearly visible. Include numbers along axes only for bottom left plot of group (a 'group' is an analysis of identical markers).

☒ All plots are contour plots with outliers or pseudocolor plots.

☒ A numerical value for number of cells or percentage (with statistics) is provided.

Methodology

Sample preparation	This part is described in detail in the materials and methods section.
Instrument	Analyses and cell sorting were respectively performed on LSR II and ARIA flow cytometers (Becton Dickinson).
Software	FlowJo V10.4.2 and BD FACSDIVA softwares
Cell population abundance	Purity of post-sort cellular fractions is regularly analysed (reanalysis by flow cytometry according to cell markers used for sorting) and is >95%
Gating strategy	The gating on spermatogenic cellular population in C57B6N mice has been previously described in Corbineau et al., 2017 as referred in the manuscript. An example of gating strategy was not included but will be added upon revision.

☒ Tick this box to confirm that a figure exemplifying the gating strategy is provided in the Supplementary Information.
